# 

**Published:** 1865-12

**Authors:** 


Volume XXII. Number 12.

### THE CHICAGO
### MEDICAL JOURNAL

De LASKIE MILLER, M. D.,
EFHRAIM INGALS, M. D.,
Editors and Proprietors.

DECEMBER, 1865.

CHICAGO:
J. C. W. BAILEY, BOOK AND JOB PRINTER, 164 CLARK STREET.
1865.

